# Huge maxillary metastasis of an aggressive Pancoast tumor –A case report

**DOI:** 10.22088/cjim.10.3.351

**Published:** 2019

**Authors:** Ramin Forooghi, Mohammad Ranaei, Fatima Bijani, Safoura Seifi, Daryoosh Moslemi, Mohammadreza Mohammadi, Nima Nikafshar

**Affiliations:** 1 Oral Health Research Center, Health Research Institute, Babol University of Medical Sciences, Babol, Iran.; 2 Cellular and Molecular Biology Research Center, Health Research Institute, Babol University of Medical Sciences, Babol, Iran.; 3 Student. Research Committee, Babol University of Medical Science, Babol, Iran.; 4 Oral Health Research Center, Institute of Health, Babol University of Medical Sciences, Babol, Iran.; 5 Cancer Research Center, Health Research Institute. Babol University of Medical Sciences, Babol, Iran

**Keywords:** Metastatic carcinoma, Pancoast tumor, Gingival metastasis, Immunohistochemical staining.

## Abstract

**Background:**

Metastatic carcinomas to the upper jaw region are very rare and unfortunately occur in advanced stages of malignancies. Pancoast tumor is a challenging subset of lung carcinoma commonly followed by distant metastasis. Since the metastatic lesion of our patient was very huge and unusual, we decided to report the case.

**Case Presentation:**

Our patient was a middle-aged heavy smoker male with a history of unresectable pancoast tumor. He was referred to the dental clinic with an expanded maxillary metastasis involving the bone and sinus region as well as oral soft tissues. To confirm the primary site of his malignancy, immunohistochemical staining was performed.

**Conclusion:**

Distant metastases of a pancoast tumor are more frequent when the primary tumor is unoperable and bone involvement is one of the early manifestations of disease.

According to the literature, about 5% of malignancies involve the oral cavity. Metastatic tumors of the jaws and oral cavity are infrequent and almost 1% of all oral tumors are metastases from other parts of the body (1). Among them, the jaw bones are more probable to be involved than oral soft tissues (2) which are accounted nearly 0.1% of all metastatic tumors (3). Gingiva and alveolar mucosa followed by tongue are the most common sites for oral soft tissue metastases (3, 4). The primary sites of cancers are breast, lung and kidney respectively (4). On the other hand, lung cancer is one of the most common malignancies and also the main cause of death due to cancer. The rate of mortality from lung malignancies is reported to be about 90% (5). Pancoast tumor is a clinically unique and challenging subset of lung non-small-cell carcinoma and represents 3% to 5% of all lung cancers (6). These tumors may invade muscles, upper ribs, thoracic vertebral bodies, subclavian vessels, the inferior portion of the brachial plexus, and the upper end of the thoracic autonomic chain including the stellate ganglion (7). Most oral metastases present a bony swelling with tenderness, pain, paresthesia, ulcer, hemorrhage and sometimes pathologic fracture, tooth mobility and trismus. Nonaggressive appearance in soft tissue resembling reactive or benign lesions might be seen in some cases. Such features can mislead the clinicians to discern the lesion as an odontogenic infections (4). Radiographically, a high percentage of maxillofacial metastatic lesions are radiolucent. Some others have mixed radiolucent-radiopaque feature, while some of them do not show any radiographic changes. Metastatic carcinomas originated from lungs are commonly adenocarcinomas followed by squamous cell carcinoma. The minority of patients are diagnosed as small cell carcinoma (1).

Definite diagnosis of metastatic tumors in maxillofacial region is very challenging and to confirm the site of the primary lesion immunohistochemical staining is necessary especially when the metastasis is the first presentation of disease (8). Less than 50% of patients with Pancoast tumor have resectable lesions because they usually show vertebral involvement or distant metastasis (6). Metastatic lesions are mostly managed by chemotherapy, radiotherapy or surgical excision (8). The prognosis of patients with distant metastases is not good and it is the main cause of death in those patients (1). The survival rate in gingival metastasis is reported from a few weeks to one year, with a maximum time of 5 years (3). In this article, we are determined on reporting a case of Pancoast tumor with a huge maxillary metastasis involving both bony and soft tissue.

## Case Presentation

**Fig 1:**
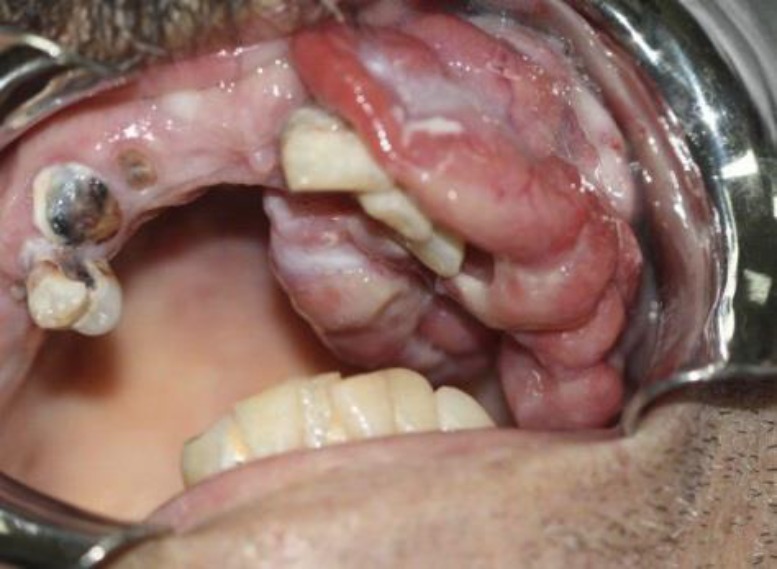
Intraoral view showing huge exophytic mass with lobulated surface

In December 2017, a 57 year-old male was referred to the Oral Medicine Department of Dental Faculty of Babol University of Medical Sciences. The chief complaint was a massive and rapidly growing swelling in his left maxillary alveolar ridge after tooth extraction a month before. The lesion was a sessile exophytic mass with lobular surface which was erythematous in some areas. He also complained from spontaneous bleeding and tenderness. The whole lesion measurements were approximately 5cm x 5cm. The involved teeth including left maxillary canine and lateral incisor had mobility grade III (fig 1). 

**Fig 2:**
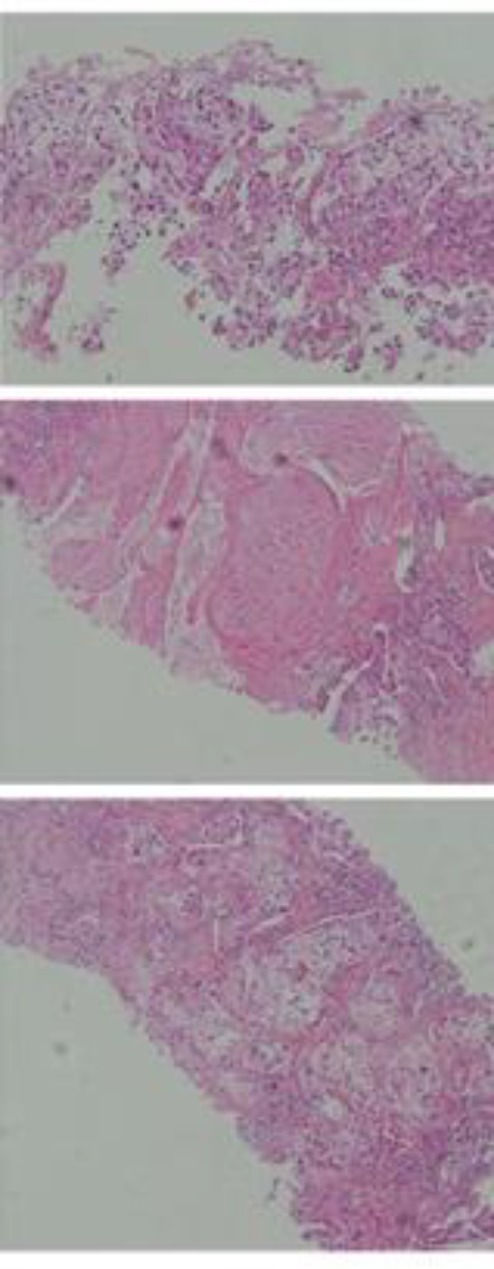
Needle biopsy from lung (Hematoxylin & Eosin)

Based on his own statements, the patient was considered as a heavy smoker who had been smoking for a long time and reported a history of having a lung malignancy since December 2016. A complete evaluation of his medical history was performed by exploring the documents of his previous hospitalization. First, after consulting with a lung specialist, a core needle biopsy from right lung mass was done and microscopic examination of sections revealed harboring neoplastic proliferation of epithelial cells with sheet and alveolar pattern. Neoplastic cells had large hyperchromatic nuclei, clear to granular eosinophilic cytoplasm and prominent nucleoli. Inflammatory cell infiltration and multifocal necrosis were observed (fig 2). Immunohistochemistry showed strong positive reaction to CK7 and there was no response to CK20, TTF1 and CDX2. The pathologist determined it as non-small-cell carcinoma, probably TTF1 negative lung carcinoma. 

**Fig 3:**
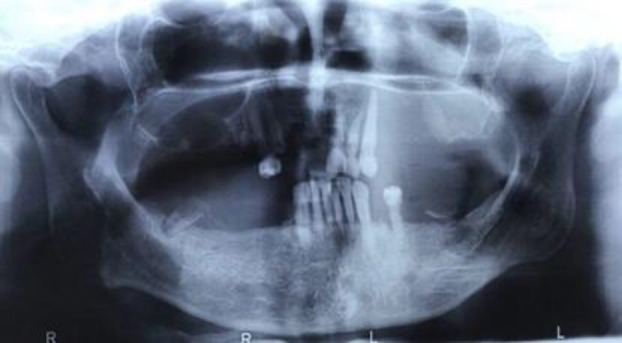
Panoramic radiograph showing maxillary left sinus involvement

No remarkable finding was shown by brain MRI taken in January 2017. Whole body bone scan was performed which showed abnormal increased uptake in the right first rib. The scan pattern suggested either a lung tumoral mass with the involvement of the first rib or radiotracer uptake by the soft tissue tumoral mass only (without bone involvement), therefore CT scan was recommended. The rest of skeleton was unremarkable. In February 2017, spiral CT of chest without contrast showed a mass lesion with ill-defined margins in the right lung apex with a maximum diameter of 62x30 millimeters in favor of a malignant process. Diffuse bilateral patchy nodular densities were noted in both lungs. There was no evidence of pleural effusion and chest wall was normal. According to all of those diagnostic procedures, the final diagnosis was unresectable Pancoast tumor. Therefore, the patient was referred to oncology service where appropriate treatment was commenced utilizing chemo-radiotherapy followed by adjuvant chemotherapy with cisplatin. Exploring the panoramic view disclosed an ill-defined radiolucency in the left maxillary area extending superiorly to the orbital floor together with cortical perforation of the alveolar ridge ipsilaterally. Left maxillary canine showed floating in air appearance, and widening of periodontal ligament was obvious in left incisors (Fig 3).

**Fig 4:**
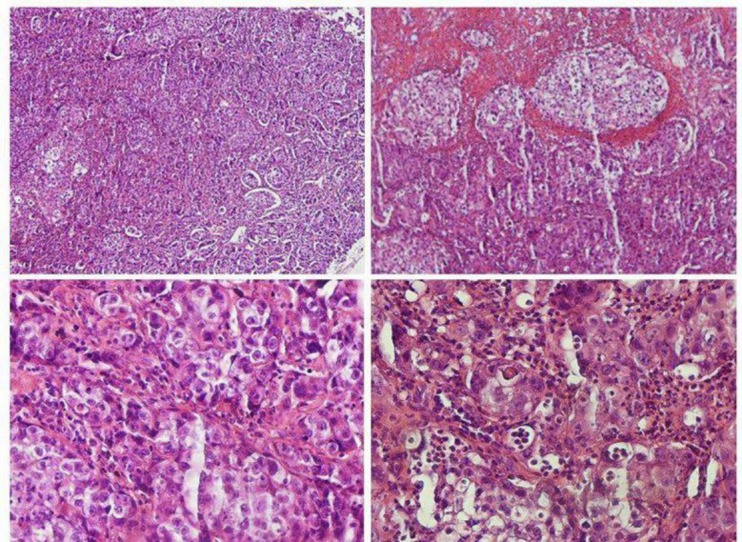
Microscopic slides of tumoral tissue stained with Hematoxylin & Eosin

Numerous specimens were taken from different parts of the lesion for histopathologic examination. Microscopic slides showed malignant neoplastic proliferation of polygonal cells in sheets and islands pattern. Most of tumoral cells had atypical hyperchromatic nuclei, and many clear cells were observed within tumoral sheets. Cellular and nuclear pleomorphism, abundant atypical mitoses, vascular invasion and large multifocal necrosis were seen (fig 4).

**Fig 5:**
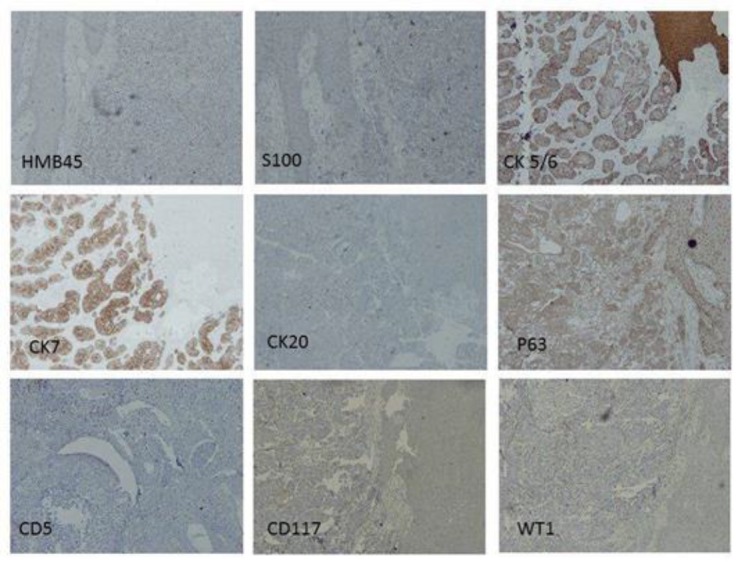
Immunohistochemical stains of tumoral tissue

According to patient’s medical history, metastatic nature of the lesion was almost decisive, but because of existing undifferentiated malignant cells, immunohistochemistry staining was recommended to confirm the origin of metastasis. Since there was no reaction to HMB45 and S100 protein, melanoma and neural crest tumors were excluded. The response to CK 5/6 was strongly positive and also positive reaction to CK7 was noticed but no response to P63, CK20, CD5, CD117, WT1 and EBV were observed (fig 5).Considering the whole findings, the metastatic lesion was designated as a squamous cell carcinoma with pulmonary origin. The consultant maxillofacial surgeon determined on not performing a surgical intervention not only because the jaw lesion did not seem to be operable due to its enormous size, but basically for the primary site of tumor which had not been eradicated appropriately. Therefore the patient was referred back to oncologist to give his advice concerning the proptitious options of chemotherapy. As the tumor was in stage IV, it was declared that chemotherapy might exacerbate the patient’s general condition. So symptomatic treatment was opted just for relieving the probable excruciating pain. Three months after commencing the case evaluation, the patient was still alive and no progression of the disease was noted.

## Discussion

The main cause of cancer related death around the world is lung cancer (5). According to literature, only 1% of all malignancies in oral cavity are metastases from distant parts (1). Majority of patients reported with metastases in oral region like this presented case were men in their 50s (1, 2, 3, 8). But some studies showed a tendency in females (1, 2) while others reported the occurrence of bony metastasis in both sexes equally and higher prevalence of soft tissue metastases in men (4). In the study of Ravi Prakash et al. metastasis to maxillary and mandibular soft tissue originated from lung cancer was only reported in men (3).

Many authors reported the breast, lung, kidney and prostate respectively as most common primary tumors for jaw metastases, but Lee et al. stated that lung and breast were the most common sources and lung was especially the first primary site in male patients (1). In other studies, lung was also the first origin for oral soft tissue metastasis (2, 3). It was compatible with Hirshberg’s study which reported the lung as the most common primary site for jaw metastasis (2). All of them showed high potential of metastasis in lung malignancies and it also happened in our case.

In more than 30% of patients with lung cancers, the invasion to bones is reported, similar to our case which was initially diagnosed. But distant metastasis to maxillary and mandibular bones is less frequent (5). Many authors reported the mandible as the first area of metastasis in oral region (1, 2) contrary to our case in maxillary region. In contrast, Ates et al. reported the case similar to our patient with maxillary soft tissue metastasis (9). Some authors stated that metastasis to oral soft tissue might resemble a nonaggressive and reactive lesion, but others rapidly reported growing exophytic masses, severe pain in teeth and jaw bones, tooth mobility and gingival bleeding as clinical presentations of metastatic tumors (1, 4, 10). Our patient showed invasive features in his maxillary hard and soft tissues.

It is suggested that chronically inflamed areas of oral mucosa particularly gingiva for its rich capillary network are the most vulnerable parts of oral soft tissue for metastasis (8). In this case, the first presentation of metastasis was noticed after tooth extraction in infected alveolar mucosa similar to the cases reported by others in literature (1).

Smoking can be considered as a risk factor of lung malignancies (11) and also may interfere with antineoplastic drugs (5). Our patient was a known heavy smoker which can justify his aggressive malignant and metastatic lesions. Prognosis of the Pancoast tumor is commonly poor and it will be aggravated especially when primary tumor can not be entirely eradicated or adjacent bony structures are invaded (12). Metastasis to oral cavity is also a manifestation of advanced stage of disease and poor prognosis (4). Accordingly, such patient is categorized as an end-stage case. Resection of the metastatic lesion can be done if possible (8), but palliative treatment including chemotherapy and radiotherapy or symptomatic treatment to reduce patient’s pain and discomfort are usually recommended (1). Our patient was in advanced stage of disease and both primary and metastatic tumors were inoperable. Moreover, it seemed that chemotherapy and radiotherapy could not help him and might worsen his condition, so we decided to follow him only by prescribing sedatives and analgesics to relieve his pain and discomfort.

Conclusion Distant metastases of a Pancoast tumor are more frequent when the primary tumor is unoperable and bone involvement is one of the early manifestations of disease.
